# Regulatory Effects of Quercetin on M1/M2 Macrophage Polarization and Oxidative/Antioxidative Balance

**DOI:** 10.3390/nu14010067

**Published:** 2021-12-24

**Authors:** Cheng-Fang Tsai, Guan-Wei Chen, Yen-Chang Chen, Ching-Kai Shen, Dah-Yuu Lu, Liang-Yo Yang, Jia-Hong Chen, Wei-Lan Yeh

**Affiliations:** 1 Department of Medical Laboratory Science and Biotechnology, Asia University, Taichung 413305, Taiwan; 2 Institute of New Drug Development, China Medical University, Taichung 404328, Taiwan; m860121@gmail.com (G.-W.C.); daniel03314@gmail.com (Y.-C.C.); 3 Graduate Institute of Biomedical Sciences, China Medical University, Taichung 404328, Taiwan; b0953295012@gmail.com; 4 Department of Pharmacology, School of Medicine, College of Medicine, China Medical University, Taichung 404328, Taiwan; dahyuu@mail.cmu.edu.tw; 5 Department of Photonics and Communication Engineering, Asia University, Taichung 413305, Taiwan; 6 Department of Physiology, School of Medicine, China Medical University, Taichung 404328, Taiwan; yangly@cmu.edu.tw; 7 Laboratory for Neural Repair, China Medical University Hospital, Taichung 404327, Taiwan; 8 Biomedical Technology R&D Center, China Medical University Hospital, Taichung 404327, Taiwan; 9 Department of General Surgery, Taichung Tzu Chi Hospital, Buddhist Tzu Chi Medical Foundation, Taichung 427213, Taiwan; guns5150@ms27.hinet.net; 10 Department of Biochemistry, School of Medicine, China Medical University, Taichung 404328, Taiwan; 11 Department of Biological Science and Technology, China Medical University, Taichung 404328, Taiwan

**Keywords:** inflammation, oxidative stress, quercetin, homeostasis, macrophage, microglia

## Abstract

Macrophage polarization plays essential and diverse roles in most diseases, such as atherosclerosis, adipose tissue inflammation, and insulin resistance. Homeostasis dysfunction in M1/M2 macrophage polarization causes pathological conditions and inflammation. Neuroinflammation is characterized by microglial activation and the concomitant production of pro-inflammatory cytokines, leading to numerous neurodegenerative diseases and psychiatric disorders. Decreased neuroinflammation can be obtained by using natural compounds, including flavonoids, which are known to ameliorate inflammatory responses. Among flavonoids, quercetin possesses multiple pharmacological applications and regulates several biological activities. In the present study, we found that quercetin effectively inhibited the expression of lipocalin-2 in both macrophages and microglial cells stimulated by lipopolysaccharides (LPS). The production of nitric oxide (NO) and expression levels of the pro-inflammatory cytokines, inducible nitric oxide synthase (iNOS) and cyclooxygenase (COX)-2, were also attenuated by quercetin treatment. Our results also showed that quercetin significantly reduced the expression levels of the M1 markers, such as interleukin (IL)-6, tumor necrosis factor (TNF)-α, and IL-1β, in the macrophages and microglia. The M1 polarization-associated chemokines, C–C motif chemokine ligand (CCL)-2 and C-X-C motif chemokine ligand (CXCL)-10, were also effectively reduced by the quercetin treatment. In addition, quercetin markedly reduced the production of various reactive oxygen species (ROS) in the microglia. The microglial phagocytic ability induced by the LPS was also effectively reduced by the quercetin treatment. Importantly, the quercetin increased the expression levels of the M2 marker, IL-10, and the endogenous antioxidants, heme oxygenase (HO)-1, glutamate-cysteine ligase catalytic subunit (GCLC), glutamate-cysteine ligase modifier subunit (GCLM), and NAD(P)H quinone oxidoreductase-1 (NQO1). The enhancement of the M2 markers and endogenous antioxidants by quercetin was activated by the AMP-activated protein kinase (AMPK) and Akt signaling pathways. Together, our study reported that the quercetin inhibited the effects of M1 polarization, including neuroinflammatory responses, ROS production, and phagocytosis. Moreover, the quercetin enhanced the M2 macrophage polarization and endogenous antioxidant expression in both macrophages and microglia. Our findings provide valuable information that quercetin may act as a potential drug for the treatment of diseases related to inflammatory disorders in the central nervous system.

## 1. Introduction

Macrophages have been categorized into at least two main polarization phenotypes: M1-polarized macrophages and M2-activated macrophages [1]. In addition, M1-type activation leads to the production of pro-inflammatory cytokines and reactive oxygen species (ROS), and M1-activated macrophages are correlated with the upregulation of ROS, which triggers macrophage activation and proinflammatory cytokine expression [2]. Conversely, the activation of M2-polarized macrophages produces neurotrophic factors [3] and secretes anti-inflammatory cytokines, such as interleukin (IL)-10, which leads to anti-inflammatory effects [4,5]. M2-macrophage polarization plays a central role in infection by limiting the immune response to pathogens, thereby preventing damage to the host [6]. In addition, chemokines, C–C motif chemokine ligand (CCL)-2 [7] and C-X-C motif chemokine ligand (CXCL)-10 [8], have been reported to be associated with Th1 immune responses. A recent report also found that the induction of CCL2 and CXCL10 secretions in macrophages exaggerated the host-derived immune responses [9]. Moreover, the heterogeneity of macrophage polarization plays a critical role in several diseases, such as atherosclerosis [10], pulmonary infection [11], and obesity-associated metabolic diseases [12]. Proinflammatory M1 macrophages also elevated the secretion of chemokines, which are thought to recruit leukocytes to areas of inflammation [13].

In the central nervous system (CNS), the microglia are the resident macrophages that are pivotal for maintaining immune defense [14]. Uncontrolled microglial activation boosts neuroinflammatory reactions and triggers neuronal death, which are pathological features of various neurodegenerative diseases [5,15] and cognitive dysfunction [4,16]. Under the influence of either a proinflammatory or anti-inflammatory microenvironment, the activated microglia polarize into two major types, M1-inflammatory or M2-anti-inflammatory phenotype [17]. Microglial polarization to the M1 state produces proinflammatory mediators, thus contributing to tissue inflammation, which may lead to neurodegenerative [18] and psychiatric disorders [4,5]. Activated microglial cells strongly elicit the phagocytosis of debris, preventing secondary inflammatory responses and promoting tissue regeneration [19]. Our previous study showed that elevated M2 macrophage genes expressions by natural products [20] or chemical compounds [21] effectively reduced the neuroinflammatory response. Therefore, developing compounds to regulate the shift in M1/M2 polarization has been suggested as a beneficial therapeutic strategy for neurological diseases [22,23].

Increasing evidence has shown that lipocalin-2 (also known as neutrophil gelatinase-associated lipocalin; NGAL) regulates various pathophysiological conditions. Previously, we analyzed the cytokine array and found that lipocalin-2 was upregulated in zymosan (derived from yeast cell wall)-stimulated alveolar macrophages [24]. Moreover, the macrophages from lipocalin-2 knockout mice display less pro-inflammatory and increased anti-inflammatory protein expression [25]. Urinary lipocalin-2 has been suggested as a risk factor for chronic kidney disease progression [26]. Remarkably, lipocalin-2 neutralization enhanced the expression of M2-related genes in a mouse cardiac ischemia-reperfusion injury model [27]. Importantly, lipocalin-2 has also been recognized as an amplifier of M1-polarization in microglial cells [28]. Furthermore, neuroinflammation-associated impairment of motor function and cognitive behavior was also diminished in the lipocalin-2-deficient mice [28]. Lipocalin 2 in the CNS is primarily released under inflammatory conditions and enhances morphological transformation and cell migration [29]. Importantly, the lipocalin-2-deficient mice showed suppressed M1 activated macrophages and enhanced the M2 response [28]. Our recent findings also showed that increased levels of lipocalin-2 in the CNS may contribute to astrogliosis and cognitive and behavioral changes [30], and exogenous lipocalin-2 in the brain impaired cognitive function and evoked anxiety-like behaviors in the animal models [30].

Quercetin, a natural polyphenol, is the most abundant flavonoid found in vegetables and fruits [31]. Quercetin has been reported to possess several biological properties, including antioxidative and anti-inflammatory effects [32]. Due to the antioxidative and anti-inflammatory properties of quercetin, the consumption of quercetin has been recently reported to exert potential human health benefits [33]. Notably, quercetin has been suggested to improve insulin resistance by inhibiting the synthesis and secretion of proinflammatory mediators [33]. Exogenous quercetin contributes to many physiological functions that may prevent and treat inflammatory, metabolic, and ischemic diseases [34]. Importantly, quercetin exerts neuroprotective effects in neurodegenerative diseases [35,36]. Recent reports have suggested that quercetin is a potential dietary supplement for modulating neuroinflammation and preventing various neurological disorders [33]. Using quercetin as a dietary supplement in humans was well tolerated and safe, and adverse effects have rarely been reported [34]. In addition, quercetin protects against obesity-induced hypothalamic inflammation by ameliorating microglia-regulated inflammatory responses via endogenous antioxidant enzyme induction [37]. Diet components and natural herbs express endogenous oxidative or cytoprotective enzymes that exert anti-oxidative stress, protect cells, and prevent inflammatory responses [38,39,40,41]. Moreover, both another group [42] and our previous findings [20,43,44,45] suggested that the up-regulation of endogenous antioxidative and anti-inflammatory enzymes may be a potential therapeutic strategy for neuroinflammation and neurodegenerative diseases.

The present study aimed to investigate the underlying mechanism of quercetin treatment for maintaining homeostasis in inflammatory/anti-inflammatory and oxidative/antioxidative effects. These results provide a better understanding of the role of quercetin in lipocalin-2 expression and M1/M2 polarization in macrophages and microglia. Our findings may help to develop therapeutic strategies for the treatment of neuroinflammation neurodegeneration and other inflammatory-associated diseases.

## 2. Materials and Methods

### 2.1. Materials

Primary antibodies against β-actin and phosphor-Akt^Ser743^ were purchased from Santa Cruz Biotechnology (Santa Cruz, CA, USA). Antibody against phospho-AMPK^Thr172^ was obtained from Cell Signaling Technology. Primary antibody against iNOS (610431) was purchased from BD Transduction Laboratories (Lexington, KY, USA). Primary antibody against COX-2 (aa 570–598) was obtained from Cayman Chemicals (Ann Arbor, MI, USA). Antibody against HO-1 was purchased from Enzo Life Sciences Inc. (Farmingdale, NY, USA). Antibodies against glyceraldehyde-3-phosphate dehydrogenase (GAPDH), GCLC, GCLM, and NQO1 were purchased from Abcam (Cambridge, MA, USA).

### 2.2. Cell Culture

RAW264.7 cells were maintained in Dulbecco’s Modified Eagle’s Medium (DMEM) with high glucose (4.5 g/L), 10% fetal bovine serum (FBS), and penicillin/streptomycin (100 U/mL) in a humidified incubator with 5% CO_2_ and 95% air at 37 °C. The adult mouse microglia cell line (IMG) derived from the adult brain was obtained from the Harvard School of Public Health (Boston, MA, USA). IMG cells expressing a microglial-specific marker fully recapitulates the morphological and functional features of brain microglia. Cells were cultured in DMEM with low glucose (1 g/L), 10% FBS, and penicillin/streptomycin (100 U/mL). The dosage of quercetin chosen for our study were referred to previous studies [46,47,48]. Evidence showed that low-dose quercetin is effective in exerting anti-tumor, anti-fibrotic, and delipidating effects. Therefore, we have set the quercetin treatment from 1 to 10 M. The viability of cells have been checked after indicated treatment (Figure S1.)

### 2.3. Western Blotting Analysis

Cells were lysed with RIPA buffer containing protease inhibitor cocktail for 30 min on ice. After centrifugation and colleting the supernatants, these protein samples were separated by SDS-PAGE and blots were transferred onto polyvinylidene fluoride membranes. After blocking with nonfat milk, the membranes were probed with primary antibodies overnight. After several washes with phosphate-buffered saline (PBS), the membranes were hybridized with secondary antibodies. The target proteins were visualized by enhanced chemiluminescence using Kodak X-OMAT LS film (Eastman Kodak, Rochester, NY, USA). Quantitative data were aquired by computing the densitometric values using ImageJ software (ImageJ).

### 2.4. NO Assay

As described in previous publication [49], NO production was measured by examing the presence of nitrite in the culture medium.

### 2.5. Quantitative Real-Time Polymerase Chain Reaction (PCR)

Total RNA was extracted from cultured cells using TRIzol reagent (Invitrogen, Carlsbad, CA, USA). Target mRNA levels were detected using quantitative real-time PCR by StepOne Real-Time PCR systems (Applied Biosystems, Foster City, CA, USA). 2 μg of total RNA was used for reverse transcription (RT) reaction and convertion to cDNA using the Invitrogen RT Kit. By amplifying DNA using oligonucleotide primers, quantitative real-time PCR was performed by using SYBR Green Master Mixes (Applied Biosystems™, Waltham, Massachusetts, USA). The threshold was set within the linear phase of target gene amplification to calculate the cycle number at which the transcript was detected (denoted as CT).

### 2.6. Phagocytosis Assay

The protocol for microglial phagocytosis was performed according to our previous study [21]. IMG cells were seeded into 3.5 cm culture dishes (5 × 10^5^ cells/well) and grown for 16 h at 37 °C and 5% CO_2_. After treatment with quercetin or LPS was administered for another 24 h, and the media were replaced by 1 mL of carboxylate-modified polystyrene fluorescent yellow-green latex beads (YG beads; Cat#L4655; Sigma Aldrich, Louis, MO, USA)-containing media and incubated at 37 °C for another 1 h. After several washes for removing the non-internalized beads, cells were incubated with ethylenediaminetetraacetic acid (EDTA; 2 mM in PBS) for 10 min at 4 °C. Afterwards, the cells were trypsinized, and the phagocytotic activity was quantified by flow cytometry(ACEA Biosciences, Sandiego, CA, USA).

### 2.7. Statistical Analysis

Statistical analysis was performed by using GraphPad Prism 6.0 (Graph Pad Software Inc, San Diego, CA, USA). Values are presented as mean ± standard error of the mean. Student’s *t*-test was used for assessing significance of the differences between experimental and control groups. Statistical comparisons of more than two groups were carried out using one-way analysis of variance with the Bonferroni post-hoc test. Significant differences were defined if the *p* value was <0.05.

## 3. Results

### 3.1. Quercetin Suppresses the Expression Levels of Lipocalin-2, Proinflammatory Cytokines, and M1-polarization Marker in Macrophages and Microglial Cells

**Figure 1:**
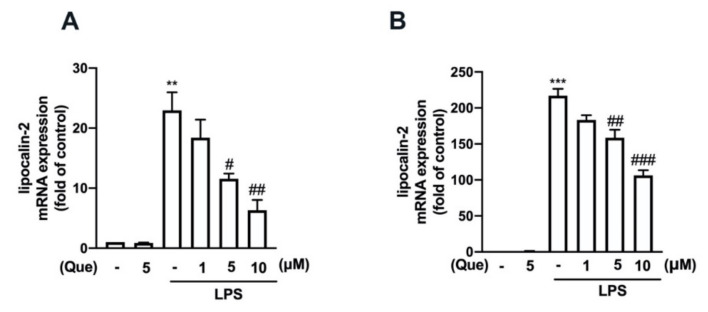
Inhibitory effects of quercetin on lipocalin-2 expression in macrophages and microglial cells. Mouse macrophage (RAW264.7) (**A**) and adult mouse microglial (IMG) (**B**) cells were stimulated with various concentrations of quercetin (1, 5, or 10 μM) for 30 min followed by stimulation with lipopolysaccharides (LPS) (50 ng/mL) for another 6 h. The mRNA levels of of lipocalin-2 were analyzed by real-time polymerase chain reaction (PCR) and normalized to β-actin. Each bar represents the mean ± standard error of the mean (SEM) (*n* = 3). Note: *** *p* < 0.005, ** *p* < 0.01 compared with the control group. ^###^ *p* < 0.005, ^##^ *p* < 0.01, ^#^ *p* < 0.05 compared with the LPS alone group.

**Figure 2:**
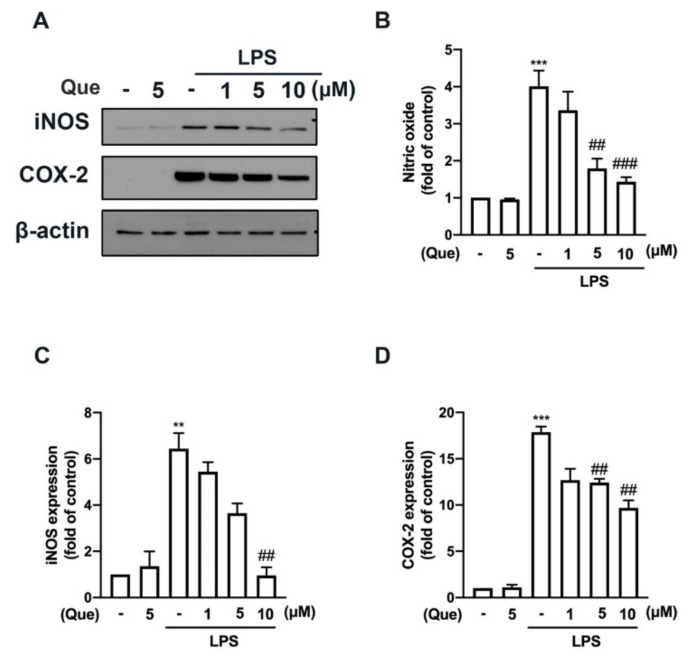
Inhibitory effects of quercetin on inducible nitric oxide synthase (iNOS) and cyclooxygenase (COX)-2 expression levels in macrophages. RAW264.7 cells were stimulated with various concentrations of quercetin (1, 5, or 10 μM) for 30 min followed by stimulation with LPS (50 ng/mL) for another 24 h. iNOS and COX-2 protein levels were determined by western blotting analysis (**A**). Quantitative results are shown in (**C**,**D**). (**B**) The supernatant was collected to determine nitric oxide (NO) production by the Griess reaction. Each bar represents the mean ± SEM of *n* = 3–4. Note: *** *p* < 0.005, ** *p* < 0.01 compared with the control group. ^###^ *p* < 0.005, ^##^ *p* < 0.01 compared with the LPS alone group.

**Figure 3:**
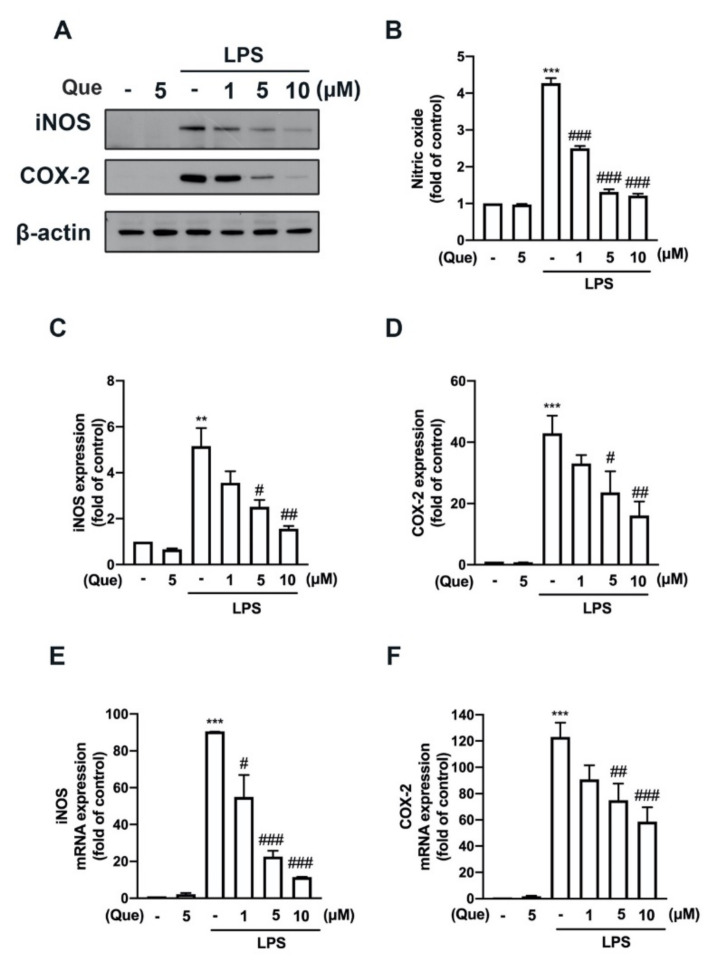
Inhibitory effects of quercetin on iNOS and COX-2 expression levels in microglial cells. IMG cells were stimulated with various concentrations of quercetin (1, 5, or 10 μM) for 30 min, followed by stimulation with LPS (50 ng/mL) for another 24 or 6 h. iNOS and COX-2 protein levels were determined by western blotting analysis (**A**). (**B**) The supernatant was collected to determine NO production by the Griess reaction. Quantitative results are shown in (**C**) and (**D**). mRNA levels of iNOS (**E**) and COX-2 (**F**) were analyzed by real-time PCR and normalized to β-actin. Each bar represents the mean ± SEM of *n* = 3–4. *** *p* < 0.005, ** *p* < 0.01 compared with the control group. Note: ^###^ *p* < 0.005, ^##^ *p* < 0.01, ^#^ *p* < 0.05 compared with the LPS alone group.

**Figure 4:**
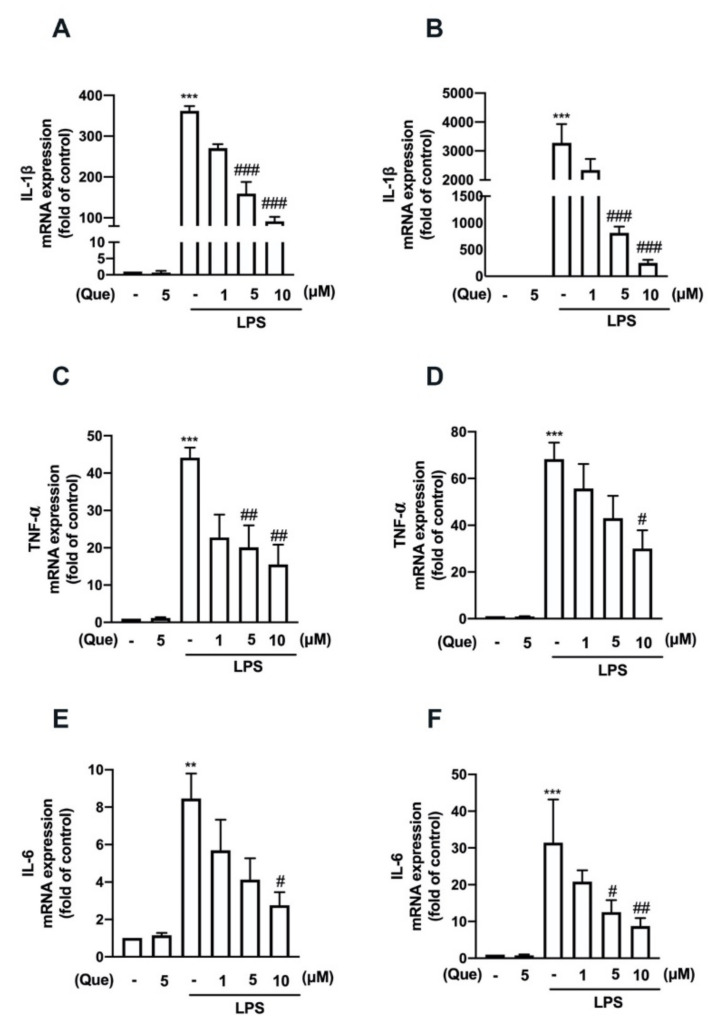
Inhibitory effects of **quercetin on** the expression of proinflammatory mediators in macrophages and microglial cells. RAW264.7 (**A**,**C**,**E**) and IMG (**B**,**D**,**F**) cells were stimulated with various concentrations of quercetin (1, 5, or 10 μM) for 30 min followed by stimulation with LPS (50 ng/mL) for another 6 h. mRNA levels of interleukin (IL)-1β (**A**,**B**), tumor necrosis factor (TNF)-α (**C**,**D**), and IL-6 (**E**,**F**) were analyzed by real-time PCR and normalized to β-actin. Each bar represents the mean ± SEM of *n* = 3–4. ^***^p < 0.005, ^**^p < 0.01 compared with the control group. Note: ^###^ *p* < 0.005, ^##^ *p* < 0.01, ^#^ *p* < 0.05 compared with the LPS alone group.

**Figure 5:**
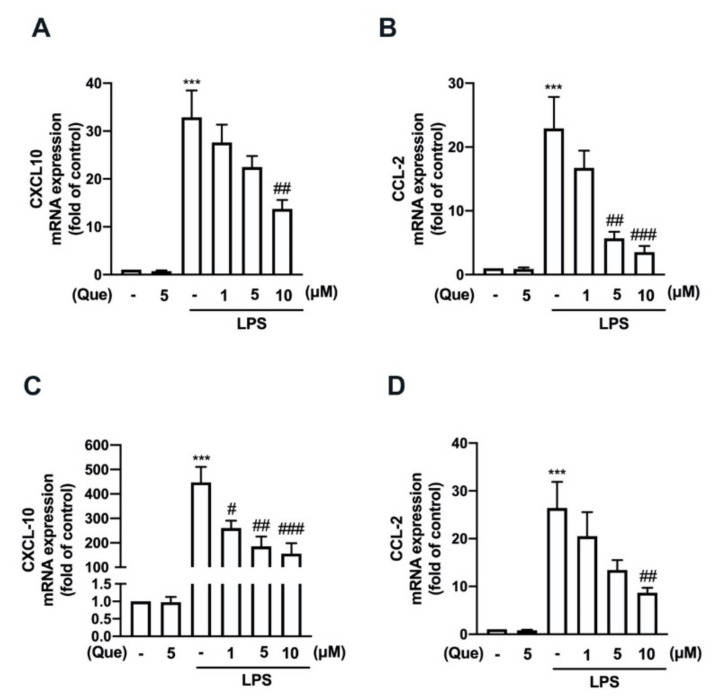
Inhibitory effects of quercetin on the expression of inflammatory mediators in macrophages and microglial cells. RAW264.7 (**A**,**B**) and IMG (**C**,**D**) cells were stimulated with various concentrations of quercetin (1, 5, or 10 μM) for 30 min followed by stimulation with LPS (50 ng/mL) for another 6 h. mRNA levels of C-X-C motif chemokine ligand (CXCL)-10 (**A**,**C**) and C–C motif chemokine ligand (CCL)-2 (**B**,**D**) were analyzed by real-time PCR and normalized to β-actin. Each bar represents the mean ± SEM (*n* = 3). Note: *** *p* < 0.005 compared with the control group. ^###^ *p* < 0.005, ^##^ *p* < 0.01, ^#^ *p* < 0.05 compared with the LPS alone group.

The mouse macrophage (RAW26.7) and adult mouse microglial (IMG) cells were used to study the anti-neuroinflammatory mechanisms of quercetin. As shown in Figure 1, stimulation with lipopolysaccharides (LPS) significantly increased the lipocalin-2 expression in both the macrophages (Figure 1A) and microglial cells (Figure 1B). In addition, the treatment with quercetin abrogated LPS-induced lipocalin-2 expression in a concentration-dependent manner (Figure 1A,B). Moreover, the treatment with quercetin also inhibited inducible nitric oxide synthase (iNOS) (Figure 2A,C) and the cyclooxygenase (COX)-2 (Figure 2A,D) protein expression levels induced by LPS in macrophages in a concentration-dependent manner. The quercetin treatment significantly reduced the LPS-induced nitric oxide (NO) production (Figure 2B). In adult mouse microglial cells, the treatment with quercetin significantly inhibited the expression of iNOS (Figure 3A,C) and COX-2 (Figure 3A,D). The enhancement of NO production was also reduced by the quercetin treatment in the microglial cells (Figure 3B). Moreover, the mRNA expression of iNOS and COX-2 induced by LPS was reduced in a concentration-dependent manner by the quercetin treatment (Figure 3E,F). We further determined the effects of quercetin on the M1 polarization in the macrophages and microglial cells. Administration of quercetin significantly attenuated the M1 polarization markers, including IL-1β (Figure 4A), tumor necrosis factor (TNF)-α (Figure 4C), and IL-6 (Figure 4E), in the macrophages. In addition, our results also show that quercetin effectively antagonizes LPS-induced IL-1β (Figure 4B), tumor necrosis factor (TNF)-α (Figure 4D), and IL-6 (Figure 4F) in microglial cells. Furthermore, the enhancement of CXCL10 (Figure 5A) and CCL2 (Figure 5B) expression in the mouse macrophages was effectively reduced by the treatment with quercetin. In addition, the treatment with quercetin enhanced CXCL10 (Figure 5C), and CCL2 (Figure 5D) were also reduced in the adult mouse brain microglia. These results indicated that LPS-primed M1 phenothypes were antagonized by quercetin.

### 3.2. Quercetin Treatment Decreases the Production of Various ROS in Microglial Cells

**Figure 6:**
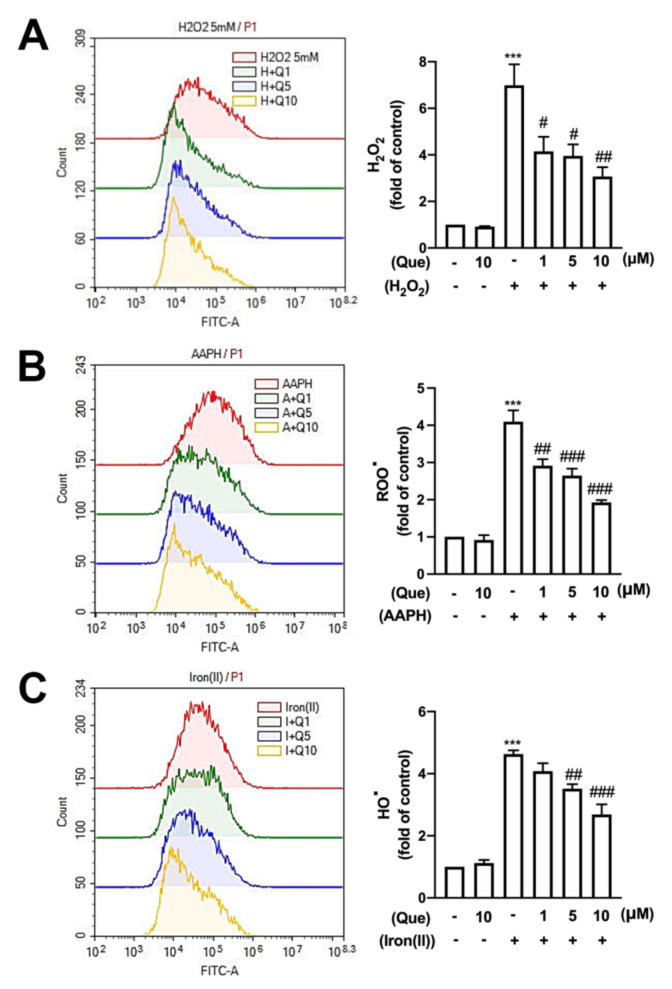
Effects of quercetin on reactive oxygen species (ROS) production in microglial cells. IMG cells were stimulated with various concentrations of quercetin (1, 5, or 10 μM) for 30 min followed by stimulation with 5 mM hydrogen peroxide (H_2_O_2_) (**A**), 5 mM 2, 2′-azobis (2-amidinopropane) hydrochloride (AAPH) (**B**), or 1 mM iron(II) plus 0.5 mM H_2_O_2_ (**C**) for another 90 min. Following incubation with 10 μM 2′, 7′-dichlorodihydrofluorescein diacetate (DCFH-DA) for 40 min, dichlorofluorescein (DCF) fluorescence intensity was detected by flow cytometry. Each bar represents the mean ± SEM (*n* = 4). *** *p* < 0.005 compared with the control group. Note: ^###^ *p* < 0.005, ^##^ *p* < 0.01, ^#^ *p* < 0.05 compared with the stimulated group alone.

To determine the effect of quercetin on the ROS production, cells were treated with hydrogen peroxide (H_2_O_2_); 2, 2′-azobis (2-amidinopropane) hydrochloride (AAPH); and iron plus H_2_O_2_, and the ROS production was determined. The ROS production was enhanced by approximately four- to six-fold (Figure 6A–C). Moreover, the administration of quercetin effectively antagonized the H_2_O_2_ production in a concentration-dependent manner (Figure 6A). The AAPH-stimulated ROOs^.^ production was also markedly inhibited by the quercetin treatment (Figure 6B). In addition, quercetin significantly reduced the iron + H_2_O_2_–increased HO^.^ production (Figure 6C). Our results showed that quercetin treatment downregulated the ROS production, such as H_2_O_2_, ROO, and HO, in a concentration-dependent manner.

### 3.3. Quercetin Reduces the Phagocytic Activity in Microglial Cells

**Figure 7:**
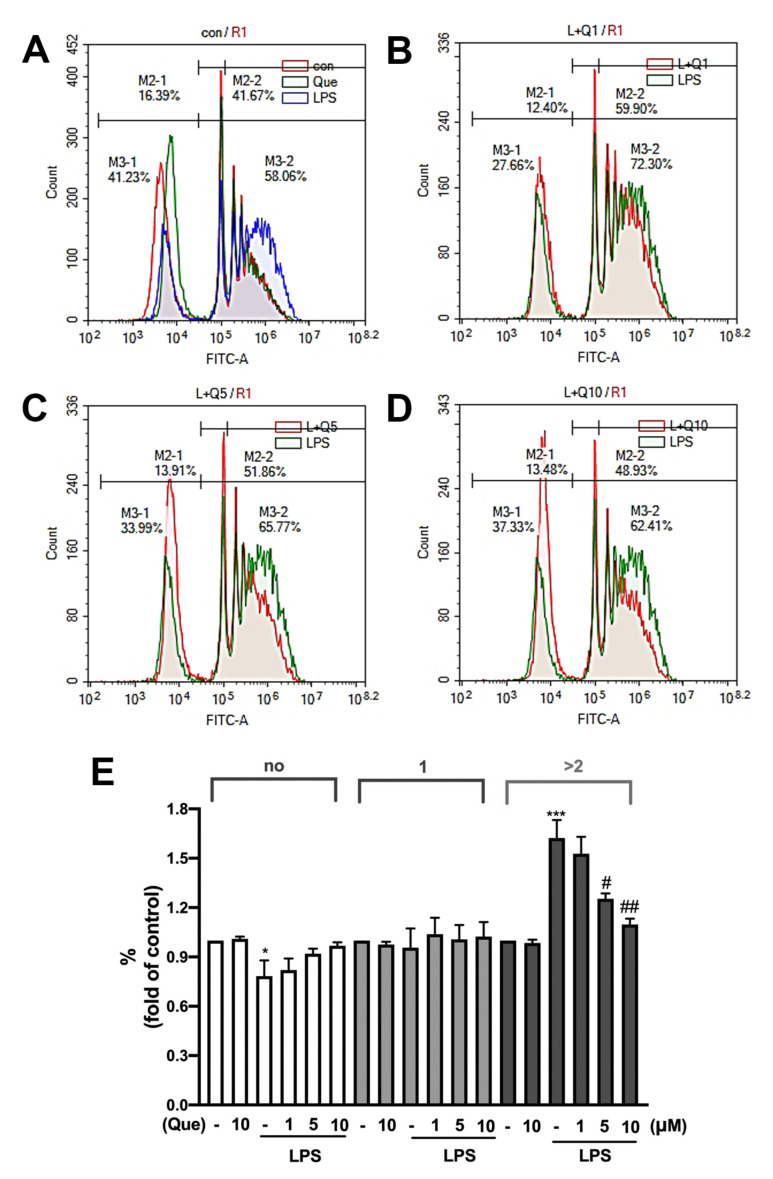
Effects of quercetin on the phagocytic ability of microglial cells. (**A**) IMG cells were stimulated either with quercetin (10 μM) or LPS (10 ng/mL) alone for 24 h. In figure B to D, following LPS (10 ng/mL) stimulation, cells were also co-treated with quercetin (1 μM in (**B**); 5 μM in (**C**); 10 μM in (**D**)) for 24 h. By monitoring the degree of fluorescence intensity by flow cytometry, populations of microglia that engulfed 0, 1, 2, or more beads can be differentiated. The quantitative results were shown in (**E**). Each bar represents the mean ± SEM of *n* = 4. Note: *** *p* < 0.005, * *p* < 0.05 compared with the control group. ^##^ *p* < 0.01, ^#^ *p* < 0.05 compared with the LPS alone group.

Next, we determined whether the quercetin treatment affected microglial phagocytosis. As shown in Figure 7A,E, the treatment with LPS significantly reduced un-phagocytic populations and enhanced the phagocytic ability of the engulfed ≥2 YG beads in microglia. In addition, the quercetin treatment alone did not affect microglial phagocytosis in either the one or >2 beads (Figure 7A,E). Furthermore, the treatment with quercetin 1 μM slightly regulated LPS-induced microglial phagocytosis (Figure 7B). Moreover, the treatment with quercetin at concentrations of 5 and 10 μM dramatically attenuated LPS-induced microglial phagocytosis by >2 beads (Figure 7C,D). These results indicated that quercetin effectively reduced microglial phagocytosis, and the treatment with quercetin alone did not affect the phagocytic ability. 

### 3.4. Quercetin Upregulates M2 Polarization and Endogenous Antioxidant Expression via the AMP-Activated Protein Kinase (AMPK) and Akt Signaling Pathways

**Figure 8:**
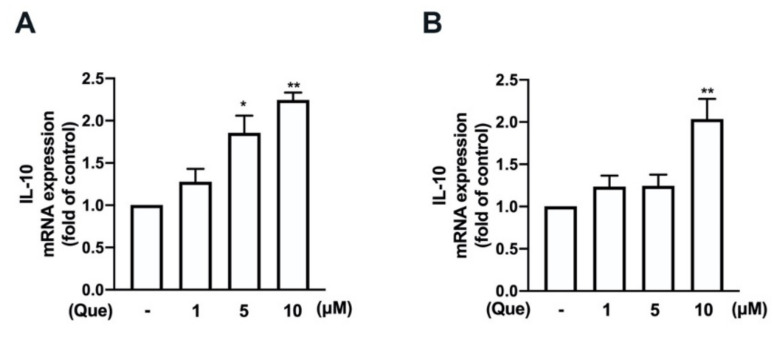
Effects of quercetin on the expression levels of anti-inflammation gene IL-10 in macrophages and microglial cells. RAW264.7 (**A**) and IMG (**B**) cells were stimulated with various concentrations of quercetin (1, 5, or 10 μM) for 6 h. The mRNA levels IL-10 were analyzed by real-time PCR and normalized to β-actin. Each bar represents the mean ± SEM (*n* = 3). Note: ** *p* < 0.01, * *p* < 0.05 compared with the control group.

**Figure 9:**
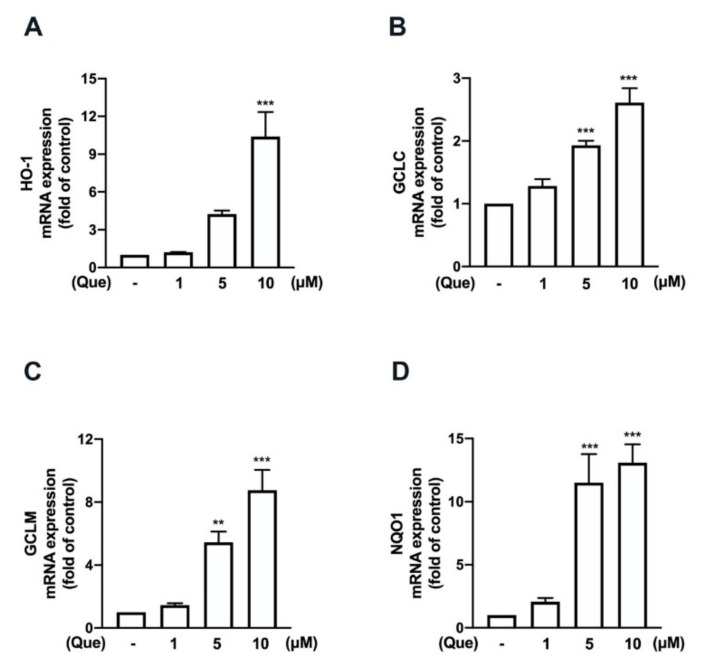
Effects of quercetin on the expression levels of endogenous antioxidants in microglial cells. IMG cells were stimulated with various concentrations of quercetin (1, 5, or 10 μM) for 6 h. mRNA levels of heme oxygenase (HO)-1 (**A**), glutamate-cysteine ligase catalytic subunit (GCLC) (**B**), glutamate-cysteine ligase modifier subunit (GCLM) (**C**), and NAD(P)H quinone oxidoreductase-1 (NQO1) (**D**) were analyzed by real-time PCR and normalized to β-actin. Each bar represents the mean ± SEM of *n* = 3–4. Note: *** *p* < 0.005, ** *p* < 0.01, compared with the control group.

**Figure 10:**
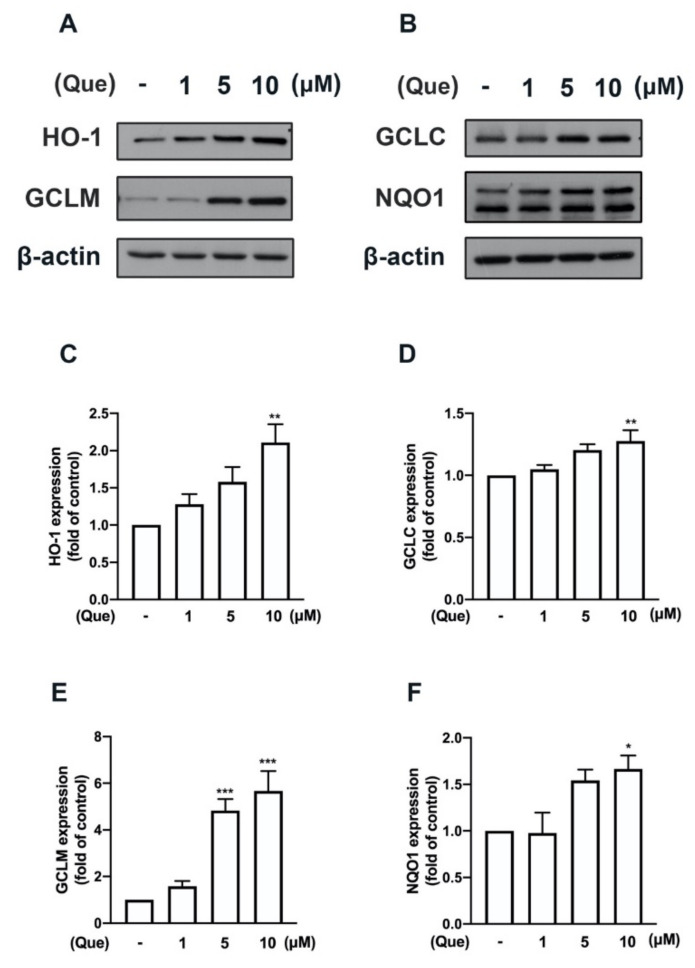
Quercetin promotes the expression of endogenous antioxidants in microglial cells. IMG cells were stimulated with various concentrations of quercetin (1, 5, or 10 μM) for 24 h. HO-1, GCLC, GCLM, and NQO1 protein levels were determined by western blotting analysis (**A**,**B**). The quantitative results of HO-1 (**C**), GCLC (**D**), GCLM (**E**), and NQO1 (**F**) were determined using ImageJ software. Each bar represents the mean ± SEM (*n* = 3). Note: *** *p* < 0.001, ** *p* < 0.01, * *p* < 0.05 compared with the control group.

**Figure 11:**
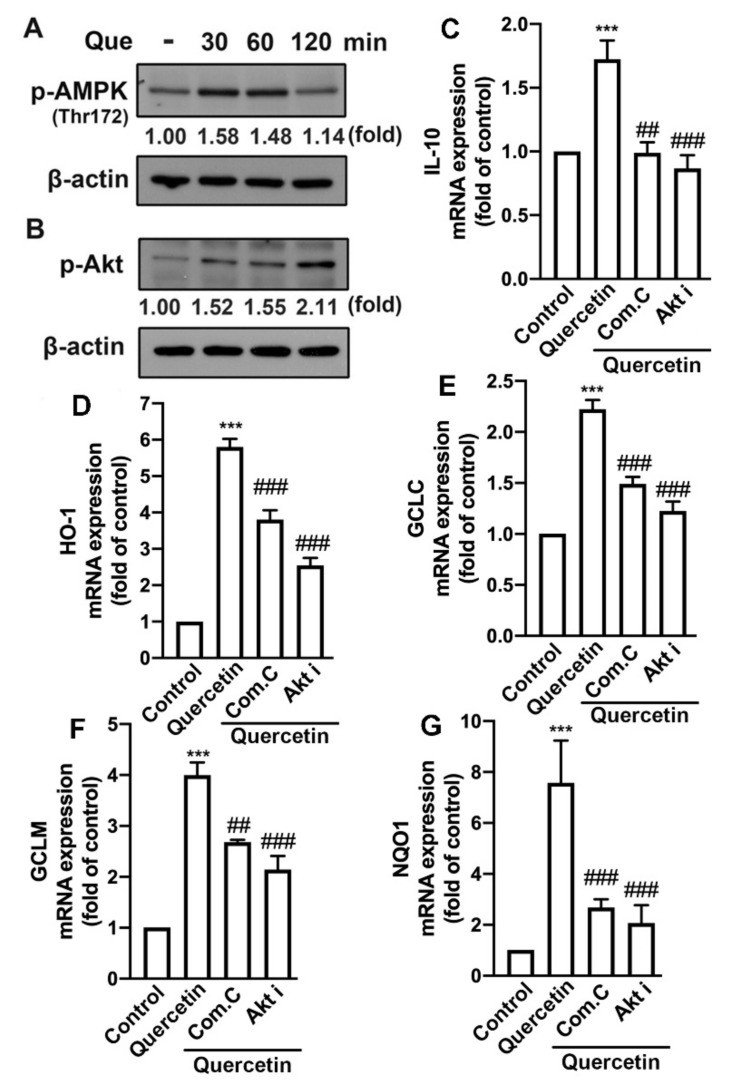
AMP-activated protein kinase (AMPK) and Akt signaling pathways are involved in the expression of quercetin-upregulated endogenous antioxidants. IMG cells were treated with quercetin (10 μM) for the indicated time periods (30, 60, or 120 min). The phosphorylated levels of AMPK (**A**) and Akt (**B**) were determined by western blotting analysis. Cells were treated with the AMPK inhibitor compound C (15 μM) or Akt inhibitor (10 μM) for 30 min and then stimulated with quercetin (10 μM) for 6 h. mRNA levels of IL-10 (**C**), HO-1 (**D**), GCLC (**E**), GCLM (**F**), and NQO1 (**G**) were analyzed by real-time PCR and normalized to β-actin. Each bar represents the mean ± SEM of *n* = 3–4. *** *p* < 0.005 compared with the control group. Note: ^###^ *p* < 0.005, ^##^ *p* < 0.01 compared with the quercetin alone group.

We have previously demonstrated that the induction of M2 polarization [21] and endogenous antioxidants [43,45] plays important roles in maintaining inflammatory/anti-inflammatory and oxidative/antioxidative homeostasis. Next, we determined the effects of quercetin on the M2 polarization in macrophages and microglial cells. In particular, the stimulation of the quercetin concentration-dependently upregulated the expression of the M2-polarized marker IL-10 in both the macrophages (Figure 8A) and microglial cells (Figure 8B). In addition, the M2 phenotype cell surface marker CD206 was also upregulated by quercetin measured by flow cytometry (Supplementary Figure S2). As shown in Figure 9, the treatment with quercetin increased the mRNA expression of endogenous antioxidants, including heme oxygenase (HO)-1, glutamate-cysteine ligase catalytic subunit (GCLC), glutamate-cysteine ligase modifier subunit (GCLM), and NAD(P)H quinone oxidoreductase-1 (NQO1), in the microglial cells. In addition, quercetin also upregulated the protein levels of these endogenous antioxidants in the microglial cells in a concentration-dependent manner (Figure 10). It has been reported that quercetin activates AMPK, which triggers anti-inflammatory responses for obesity treatment [50]. Moreover, quercetin has also been found to modulate inflammatory processes through a variety of signaling pathways, including the phosphatidylinositol-3-phosphate kinase (PI3K)/Akt signaling pathway [33]. As shown in Figure 11, the stimulation with quercetin increased the AMPK (Figure 11A) and Akt (Figure 11B) activation. Moreover, treatment with an AMPK inhibitor (compound C) and an Akt inhibitor effectively antagonized the quercetin-associated IL-10 expression (Figure 11C). In addition, the enhancement of HO-1 (Figure 11D), GCLC (Figure 11E), GCLM (Figure 11F), and NQO1 (Figure 11G) were reduced by both compound C and the Akt inhibitor. Our findings indicate that quercetin upregulates the M2 polarization and endogenous antioxidant expression through the AMPK and Akt signaling pathways.

## 4. Discussion

Recent studies have suggested that the different polarization of macrophage phenotypes contributes to metabolic homeostasis in several diseases, including obesity, atherosclerosis, insulin resistance, and cancer. M1 macrophages have been recognized to contribute to insulin resistance, whereas M2 macrophages protect against insulin resistance [51]. Adipose tissue macrophages stimulated by free fatty acids, produce CCL2 that promote the recruitment of proinflammatory monocytes to the inflamed tissue [52], but L-10 may protect adipocytes from obesity, causing insulin resistance [53]. In the CNS, M2 microglia activation has been reported to suppress the inflammatory molecules that improve hippocampal spatial learning in a mouse model [54]. In addition, it has been reported that the increased production of IL-10 exerts anti-inflammatory and analgesic effects [55]. Furthermore, IL-10 inhibits IL-6 production by preventing nuclear translocation of nuclear factor (NF)-κB in the microglia [56]. Numerous studies have also reported that quercetin has therapeutic potential for brain disorders. Treatment with quercetin has been found to have anticonvulsant activity, which correlates with its brain concentration [57]. In addition, quercetin has also been reported to contribute to antidepressive effects through the inhibition of antioxidant effects [58]. Moreover, treatment with quercetin has also been reported to attenuate hypothalamic–pituitary–adrenal (HPA) axis dysregulation in a mild traumatic brain injury mouse model [58]. Importantly, oral administration of quercetin reduced insoluble Aβ levels in the cortex of amyloid transgenic mouse models [59]. Importantly, quercetin treatment improves functional recovery after spinal cord injury by inhibiting macrophages/microglia polarization to M1, thereby protecting the cell survival of oligodendrocytes [60]. The present study showed that treatment with quercetin effectively reduced the proinflammatory and oxidative effects in the macrophages and microglial cells.

Although ROS in macrophages are essential for the uptake and clearance of dying cells, the intracellular ROS production in macrophages is thought to be involved in the phagocytic process [61]. High levels of ROS may cause macrophage apoptosis that is harmful to macrophages [62], which has been observed to increase the expression of endogenous antioxidants for adaptive survival [63]. Induction of ROS in murine macrophage RAW264.7 cell and primary peritoneal macrophages increased the phagocytic activity [64]. Interestingly, H_2_O_2_ has been observed to serve as a chemoattractant to facilitate macrophage recruitment to monocytes [65]. Moreover, ROS triggered CCL2-induced hyperalgesia, which was attenuated in the presence of SOD in a rodent model [66]. Furthermore, the mitochondria-derived ROS have also been found to be activated by IL-1β secretion [67]; the treatment with quercetin decreases the ROS production induced by IL-1β [68]. Interestingly, quercetin has also been found to suppress ROS via microbiota exerting a neuroprotective effect [69]. Importantly, oxidized LDL has been recognized to stimulate the M1 macrophage activation and further support monocyte recruitment, which may lead to atherosclerosis [70]. It is widely believed that cytoprotective protein expression exerts antioxidant effects and participates in cell protection [71]. Many endogenous antioxidants are involved in maintaining redox homeostasis and against CNS diseases, including HO-1, GCLC, GCLM, and NQO-1 [72]. Our results showed that the administration of quercetin reduced the ROS production and increased the expression of various endogenous antioxidants.

In the CNS system, the inhibition of ROS production, M1-like pro-inflammatory phenotype, and elevation of the M2-like macrophage activation IL-10 improved spinal cord injury [73]. Using immunomodulation targets, IL-10 production has been considered as a therapeutic potentiation in several neurodegenerative diseases, including Alzheimer’s disease, Parkinson’s disease, and multiple sclerosis [74]. Exogenous interleukin-10 administration decreases inflammatory extent and improves motor function after a spinal cord injury [75]. M2 microglia protect the neighboring cells by secreting trophic factors such as IL-10 for repairing brain [76]. Furthermore, the findings of HO-1 downstream of IL-10 reveals new possibilities for better therapeutic approaches for treating inflammatory diseases [77]. The protective effect of IL-10- against LPS-induced septic shock in mice was markedly attenuated by cotreating with the HO inhibitor [77]. Induction of HO-1 by natural or synthetic antioxidants drives M1 to M2 switching and improves kidney function in diabetes [78]. The anti-inflammatory effect of the antidepressant amitriptyline on morphine tolerance is regulated by HO-1 signal transduction, possibly by increasing IL-10 production [79]. In our previous report, the activation of the endogenous antioxidant enzyme HO-1 exhibits anti-neuroinflammatory effects on the microglial cells [20,43,44,45] and neuroprotection against neurotoxin-induced cell death [80]. In addition, our previous report [20,81] also demonstrated that the elevation of HO-1 expression polarizes the macrophage/microglia toward the M2 phenotype. Moreover, quercetin reduces microglia-mediated inflammatory responses in obesity through HO-1 induction [37] and protects glial cells by reducing the ROS production and inducing HO-1 expression [82]. Our results showed that treatment with quercetin markedly reduced proinflammatory cytokine expression and increased the expression of the M2 marker IL-10 in both macrophages and the microglial cells.

In the CNS system, lipocalin-2 knockout mice with cerebral ischemia have been found to have smaller infarct volumes and better neurological functions, but the unexpected activation of astrocytes was not observed [83]. Recently, lipocalin-2 has been demonstrated to play a crucial role in pathological conditions, especially in pathological progression associated with cognition [84,85]. Elevated ItI It has been reported that lipocalin-2 is upregulated in a mouse’s hippocampus under psychological stress and modulates stress-induced neuronal excitability and anxious behavior [86], which has also been considered as a potential biomarker in Alzheimer’s disease and other aging-related cognitive decline [87]. Lipocalin 2-null mice demonstrate not only anxious and depressive-like behaviors, but also cognitive and memory impairments [88]. Clinically, lipocalin-2 in cerebrospinal fluid is a convincing biomarker for the differential diagnosis between vascular dementia and neurodegenerative dementias [89]. Furthermore, single prolonged stress-enhanced activation on astrocyte in rat can last for days, augmenting the vulnerability of abnormal fear learning [90]. A recent study reported that Parkinson’s disease patients are more responsive to lipocalin-2 stimulation for reactive astrocytosis [91]. We recently reported that increased lipocalin-2 in the CNS contributes to astrogliosis as well as cognitive and behavioral dysfunction [30], and increased lipocalin-2 in the brain impaired cognitive function and evoked anxiety-like behaviors in animal models [30]. To the best of our knowledge, the present study is the first to report that treatment with quercetin significantly reduced the lipocalin-2 expression in both macrophages and microglial cells.

Phagocytosis in the central nervous system is normally exerted by the phagocytic cells, especially microglia and macrophages [92].The phagocytic removal of debris by microglia has been proposed to be of biological significance and is associated with the progression of neurological diseases, such as Alzheimer’s disease (AD) [93,94], Huntington’s disease (HD) [95] and Parkinson’s disease (PD) [96], as well as trauma and stroke [3]. Although it remains to be elucidated whether this phagocytic behavior is beneficial or detrimental during various stages of brain diseases or injury, many studies have demonstrated that the efficient clearance of debris by the microglia creates a favorable environment for subsequent reconstruction and reorganization of neuronal networking in a diseased brain [3,97]. For example, clearing the extracellular accumulation of β-amyloid (Aβ) via phagocytosis of Aβ by the activated microglia has been observed in experimental AD models [98,99,100] and modulating the activity of microglial phagocytosis might be a potential target for AD or the treatment for other brain injuries [3,100,101]. Notably, these beneficial microglial functions often involve changes in the morphology and protein expression, yet their function is distinct from that of classic pro-inflammatory responses. Activated microglia release cytokines and chemokines and execute phagocytosis activity [100]. In particular, phagocytosis has been proposed as a form of cell death caused by the phagocytosis of viable cells, resulting in their destruction [92]. The activation of microglial cells strongly elicits the phagocytosis of debris to prevent secondary inflammatory responses and tissue degeneration [19]. To the best of our knowledge, this study is the first to show the effects of quercetin on the proinflammatory stimuli-induced phagocytic ability in microglial cells.

## 5. Conclusions

Our findings indicate that treatment with quercetin effectively reduces the M1 inflammatory responses that stimulate NO production, proinflammatory cytokine expression, and lipocalin-2 production in both macrophages and microglial cells. The chemokines, CCL2 and CXCL10, were also inhibited by quercetin treatment. In addition, quercetin inhibits the production of ROS, such as H2O2, ROO, and HO. The microglial phagocytic ability induced by the proinflammatory stimuli was also reduced by the quercetin treatment; however, quercetin effectively increased the expression levels of the M2 marker, IL-10, in both the macrophages and microglial cells. Importantly, our results also showed that the treatment with quercetin upregulated a variety of endogenous antioxidants, including HO-1, GCLM, GCLC, and NQO1. Therefore, our results indicate that quercetin may be a useful therapeutic target for the treatment of neuroinflammation-associated disorders and other inflammatory-associated diseases owing to its role in the modulation of inflammatory homeostasis that could contribute to maintaining M1/M2 polarization and oxidation/antioxidation effects.

## Data Availability

Data are available from the corresponding author on reasonable request.

## Supplementary Materials

The following are available online at https://www.mdpi.com/article/10.3390/nu14010067/s1, Figure S1: Effects of quercetin on cell viability in macrophages and microglial cells, Figure S2: Quercetin induces cell surface CD206 expression on macrophages.
