# Supplementary material for: Regulatory Effects of Quercetin on M1/M2 Macrophage Polarization and Oxidative/Antioxidative Balance
Source: Nutrients. 2021 Dec 24;14(1):67. doi: 10.3390/nu14010067 (PMC8746507; doi:10.3390/nu14010067)
Supplement: Supplementary file 1 [file nutrients-14-00067-s001.zip › nutrients-1483598-supplementary.pdf]

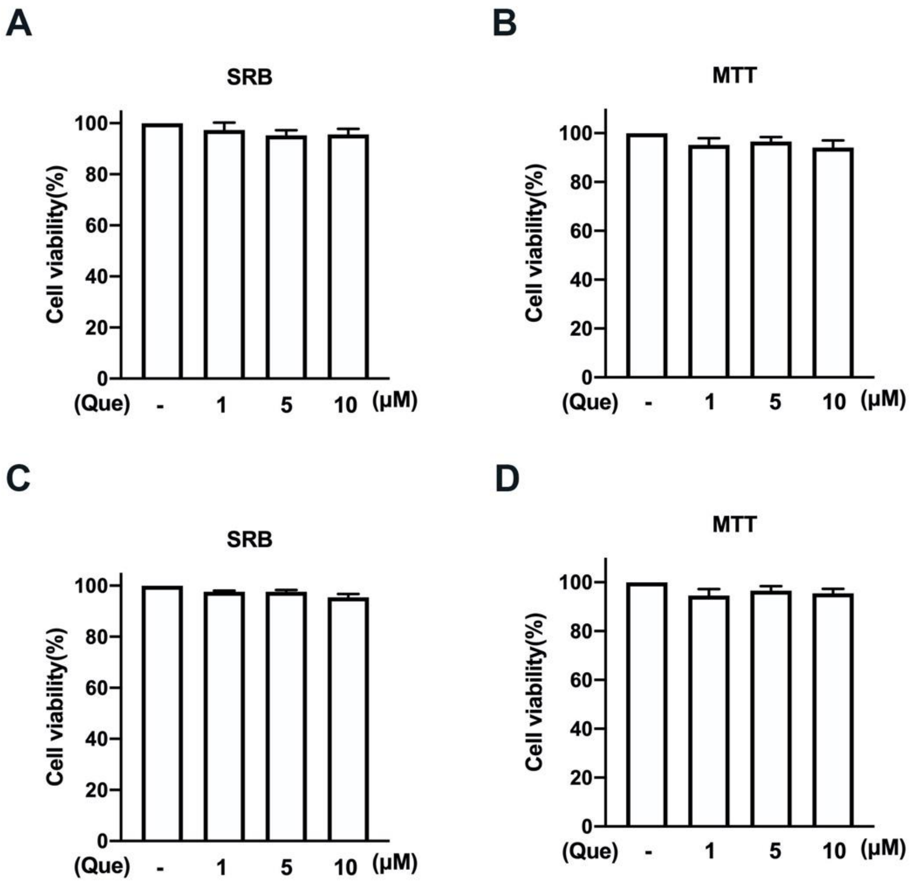


**Supplementary Figure S1. Effects of quercetin on cell viability in macrophages and microglial cells.** RAW264.7 (A, B) and IMG (C, D) cells were incubated with various concentrations (1, 5 or10 μM) of quercetin for 24 h, and then the cell viability were determined by SRB (A, C) and MTT (B, D) assays. The data represent mean ± SEM of n=4.

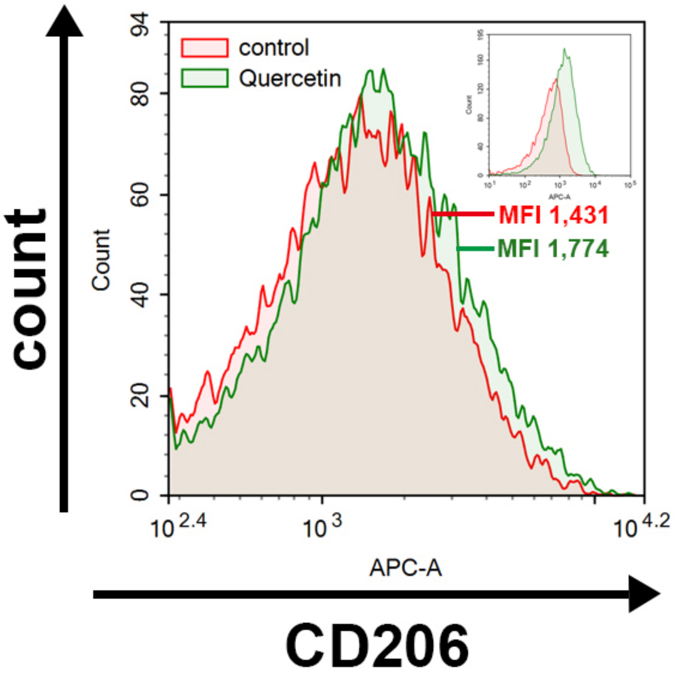


**Supplementary Figure S2. Quercetin induces cell surface CD206 expression on macrophages.** RAW264.7 cells were incubated with 10 µM of quercetin for 24 h, and the expression of cell surface M2 phenotype marker CD206 was evaluated by flow cytometry. The upper right panel shows that control group (green) was shifted from negative control (red).
